# The Utility and Limitations of CRP, ESR and DAS28-CRP in Appraising Disease Activity in Rheumatoid Arthritis

**DOI:** 10.3389/fmed.2018.00185

**Published:** 2018-08-03

**Authors:** Carl K. Orr, Aurelie Najm, Francis Young, Trudy McGarry, Monika Biniecka, Ursula Fearon, Douglas J. Veale

**Affiliations:** ^1^Dublin Academic Medical Centre, Centre for Arthritis and Rheumatic Diseases, University College Dublin, Dublin, Ireland; ^2^Molecular Rheumatology Research Group, Trinity Biomedical Sciences Institute, Trinity College, Dublin, Ireland

**Keywords:** synovium, inflammatory biomarkers, disease activity, rheumatoid arthritis, synovial biopsy

## Abstract

**Introduction:** Identifying and quantifying inflammatory disease activity in rheumatoid arthritis remains a challenge. Many studies have suggested that a large proportion of patients may have active inflammation, but normal inflammatory markers. Although various disease activity scores have been validated, most rely to a large degree on biomarkers such as CRP and ESR. In this study, we examine the utility and limitations of these biomarkers, as well as the DAS28-CRP in appraising disease activity in RA.

**Methods:** Two hundred and twenty three consecutive rheumatoid arthritis reporting knee arthralgia underwent synovial sampling of the affected knee via needle arthroscopy. The synovium was examined by microscopy with H+E staining as well as immunohistochemistry, and related to the ESR, CRP and DAS28-CRP on blood samples taken immediately before arthroscopy.

**Results:** Although a statistically significant positive correlation was observed between CRP and the level of inflammation in the biopsy retrieved (*n* = 197, rho = 0.43, CI 0.30–0.54, *p* < 0.0001), there was histological evidence of inflammation in the synovium in 49.4% of the patients who had a normal CRP. A positive correlation was also observed between ESR and the level of inflammation in the biopsy retrieved (*n* = 188, rho = 0.29, CI 0.15–0.42 *p* < 0.0001). A statistically significant but weak positive correlation was observed between the DAS28-CRP and synovial inflammation (*n* = 189, rho = 0.23, CI 0.09–0.37, *p* = 0.0011). Only the CD19 infiltrate in the synovium correlated with serum CRP (*n* = 70, rho = 0.32, CI 0.08–0.52, *p* = 0.0068).

**Conclusion:** CRP has a moderately strong relationship with disease activity, but there are significant pitfalls in the use of this biomarker in RA, and therefore a need interpret CRP results judiciously. The results of this study underline the heterogeneity of RA, and the need to develop improved panels of biomarkers, to better stratify RA, and to identify the cohort for whom inflammatory activity cannot be measured accurately with CRP.

## Introduction

Rheumatoid arthritis (RA) is a chronic, progressive autoimmune disorder characterized by synovial proliferation and degradation of articular cartilage and bone (1). It is now recognized that RA is an extremely heterogenous disease, with a tendency to chronicity, the course of which features periods of flares, as well as low disease activity or remission. Well validated disease activity scores have been developed that allow comparisons between sequential assessments be made, as well as the adoption of the treat-to-target approach which is now recommended by ACR/EULAR (2).

The various disease activity scores (DAS) comprise composite parameters, including counts of tender and swollen joints, patient global health self-report, and levels of serum C-reactive protein (CRP) or erythrocyte sedimentation rates (ESR). The most common in use is the DAS28, incorporating either CRP or ESR (3), and it had been shown that a selection of the 28 named joints is as valid and reliable as more comprehensive joint counts in clinical care and trials (4). As CRP is now widely accessible, and is responsive in a more timely manner to changes in systemic inflammatory activity, the DAS28—CRP is widely used today (5). There is very high agreement between DAS28 calculated using ESR with that calculated using CRP (6), and both are well validated. The DAS28-CRP has been shown to give an overall slightly lower score than the ESR (7–9). Some contend therefore that the DAS28-CRP underestimates disease activity, especially when cut-off values validated in DAS28-ESR are used to classify the level of activity (remission, low, moderate and high disease activity) (10). For an excellent review of disease activity indices, as well as response criteria and remission definitions, see Salomon-Escoto et al. (11).

Notwithstanding the fact that the various DAS have been well validated, assessing disease activity in the individual patient in clinic remains challenging. There are inherent confounders in each parameter of the DAS28-CRP. The joint counts are patient and examiner dependant, and are therefore subjective. The patient global health report is entirely subjective. Furthermore, the weighting within the score for a swollen knee is the same as that for a swollen PIP, despite the clearly greater inflammatory and functional burden from the former.

In relation to CRP and ESR, the only completely objective component of the DAS28 scores, it is noteworthy that RA registry data for over 9000 patients has shown that more than half did not have elevation of ESR or CRP, but had ongoing disease activity as determined by joint counts and global assessments (12). Data from other studies support this finding of normal inflammatory markers in the context of active disease (13).

Ultrasound and magnetic resonance imaging studies have also demonstrated that a significant proportion of patients (over 50%) classified by DAS28-ESR or –CRP as being in remission, have evidence of persistent synovitis, and this may explain why such patients continue to develop erosive disease (14–16).

In this study, we examine the utility and limitations of the biomarkers CRP and ESR, as well as the DAS28-CRP in appraising disease activity in RA. Since the synovium is the principal target of inflammation in RA, we study the synovium at the microscopic level, and relate CRP, ESR, and DAS28-CRP with the histological features of synovial biopsies, including specific cellular infiltrate.

## Methods

### Patient recruitment

Consecutive RA patients with knee arthralgia were recruited from outpatient clinics at the Department of Rheumatology, St Vincent's University Hospital, in Dublin. Synovial biopsy was performed by needle arthroscopy of the affected knee. All patients fulfilled classification criteria for RA (17), and provided written informed consent. Ethical approval to conduct this study was granted by St. Vincent's Healthcare Group Medical Research and Ethics Committee.

### Arthroscopy

Under local anesthetic, arthroscopy of the knee was performed using a (Karl Storz, Germany) 2.7 mm needle arthroscope as previously described (18). Clinical examination and arthroscopy were performed by CO and DJV. Both arthroscopists have extensive experience in arthroscopic guided synovial biopsy. Prior to arthroscopy, patients underwent a full clinical assessment including profile for age, sex, disease duration and use of medications. A 28 joint count for swollen and tender joints was performed. Patients completed a patient global health on a VAS (0–100 mm). Disease activity was calculated using the DAS28-CRP. The definition of remission was a DAS28-CRP < 2.6. Laboratory investigations included measurement of ACPA, rheumatoid factor, as well as CRP and ESR, using samples taken immediately before arthroscopy.

### CRP assay

CRP was measured using Roche/Hitachi Cobas CRPL3 systems, with a sensitivity range 0.3–350 mg/L (2.9–3333 nmol/L), on patient's serum immediately before arthroscopy, with values < 5 mg/L considered normal.

### Synovial tissue handling and staining

Synovial biopsies were immediately embedded in Optimal Cutting Temperature (OCT) mounting media. Seven-micrometer sections were allowed to reach room temperature, and were fixed in acetone for 10 min and air-dried. Haematoxylin and eosin (H+E) staining was applied. For immunohistochemistry, a routine three-stage immunoperoxidase labeling technique incorporating avidin-biotin immunoperoxidase complex (DAKO, Glostrup, Denmark) was used. Sections were incubated with primary mouse monoclonal anti-CD3, anti-CD4, anti-CD8, anti-CD19, anti-CD68, and anti-FVIII antibodies (DAKO, Glostrup, Denmark) at room temperature for 1 h. Color was developed in solution containing diaminobenzadine tetrahydrochloride (Sigma-Aldrich, St Louis, Missouri, USA), 0.5% H_2_O_2_ in phosphate buffered saline (pH 7.6). Slides were counterstained with haematoxylin and mounted. All slides were stained and scored under blinded conditions. For H+E staining, slides were scored for levels of inflammation over 3 ordinal categories; 0 = no inflammation, 1 = mild inflammation, 2 = moderate to severe inflammation. Slides were also analyzed using a well-established semi-quantitative scoring method ranging from 0 to 4 for levels of immunohistochemistry staining for both lining and sub lining layers (0 = no staining, 1 = < 25%, 2 = 25–50%, 3 = 50–75%, 4 = >75% staining) (19, 20). FVIII was scored by counting the mean number of blood vessels stained per high power field (magnification x20). Sections were scored by two reviewers independently, and each were blinded to the tissue source. Differences in scoring between observers were reviewed together, and consensus achieved in each instance.

### Statistical analysis

Continuous and ordinal data that are normally distributed are presented as the mean with standard deviation (SD), and non-normally distributed data are presented as the median with interquartile range (IQR). Differences between groups were analyzed using unpaired (2 tailed) *t*-tests for normally distributed data. Differences between groups with non-normally distributed data were analyzed using the Mann-Whitney U test. Categorical data are presented as the number of subjects, with percentage. Correlations were calculated using using Spearman correlation and reported as rho. Given the challenge of interpreting ordinal data over 3 groupings, one-way ANOVA and Kruskall-Wallis tests were used to determine differences across the three ordinal groupings, for normally and non-normally distributed data respectively. Statistical analysis was performed using GraphPad Prism version 6.03 for Windows (GraphPad Software, La Jolla California USA). P values less than 0.05 were considered significant.

## Results

### Patients

**Figure 1:**
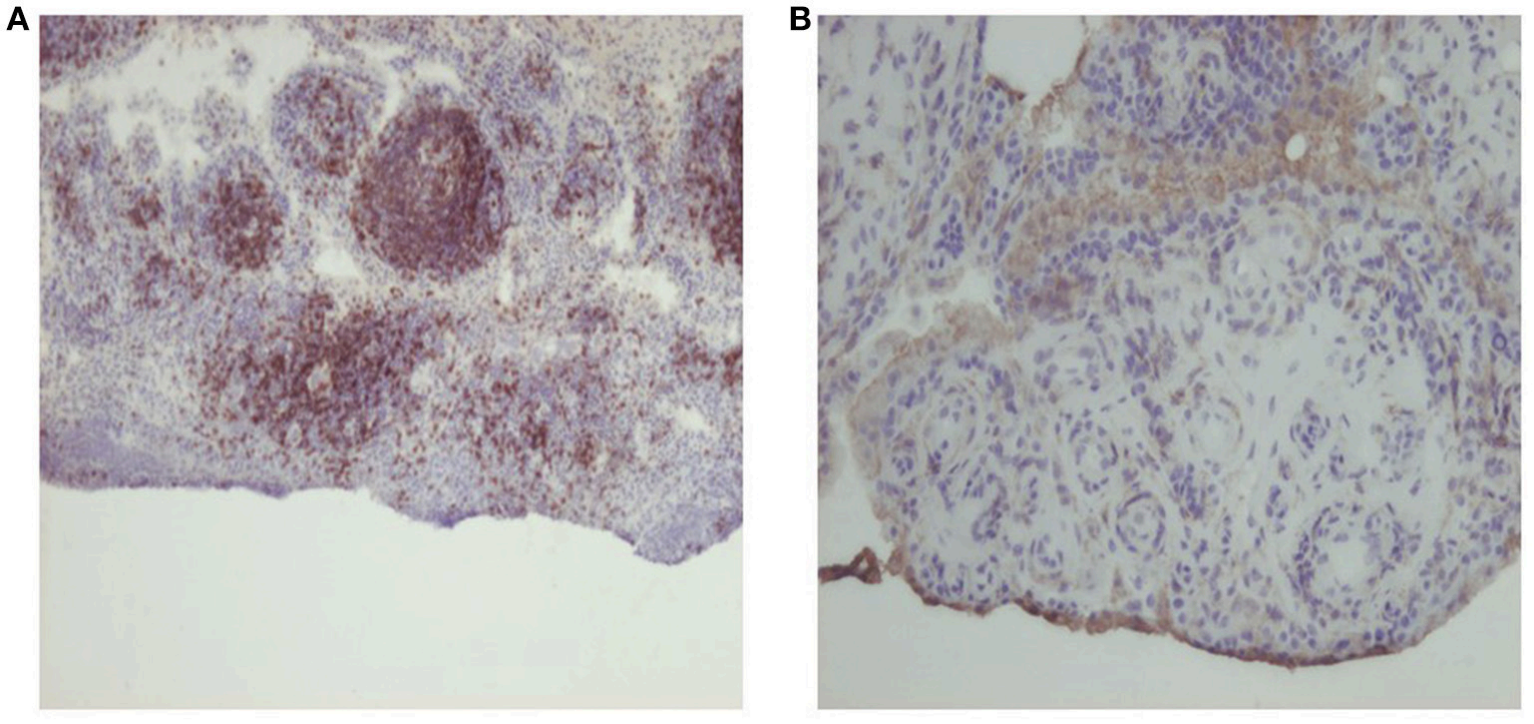
Representative photomicrographs of synovial tissue stained with haematoxylin and eosin and immunohistochemistry, for **(A)** CD3, scoring 3, original magnification 1 OX, and **(B)** CD4, scoring 1, original magnification 20X.

Two hundred and twenty three patients were included. Baseline demographics and disease characteristics were similar to other large RA cohorts and are shown in Table 1. The mean age was 54.5 (13.5), and the cohort were predominantly female (71.7%). 60.3% were positive for ACPA, and 57.0% were positive for RF. The patients were highly heterogeneous with respect to DAS28-CRP, CRP, ESR, and disease duration, and differed in treatments at the time of the arthroscopy. In this regard, the cohort fully represents the wide spectrum of RA. Exemplar images for CD3 and CD4 staining are presented in Figure 1.

**Table 1:** Demographics of cohort.

	**Total**
**CHARACTERISTIC**	
n	223
Age, years	54.5 ± 13.5
Male (%)	63 (28.3%)
RF+	127 (57.0%)
ACPA+^a^	132 (60.3%)
Disease duration, years	4 (1–13)
CRP	8.5 (3.1–26.0)
ESR^1^	23.0 (13.0-41.0)
DAS28-CRP^a^	4.395 ± 1.327
SJC^a^	3 (1–8)
TJC^a^	4 (2–9)
PGH^a^	60 (39.5–80)
Synovitis score^a^	60 (40–80)
Histology score^a^	2.0 (1.0–3.0)
**TREATMENT (%):**	
Naïve	98 (43.9%)
nbDMARD	79 (35.4%)
TNFi	36 (16.1%)
Other biologic	10 (2.7%)

a *(unknown values; ACPA 4, ESR 12, DAS28-CRP 13, SJC, and TJC 11, PGH 13, synovitis score 14, histology score 26)*.

### Outcomes

#### Relationship of CRP, ESR, and DAS28-CRP to synovial tissue inflammation

**Figure 2:**
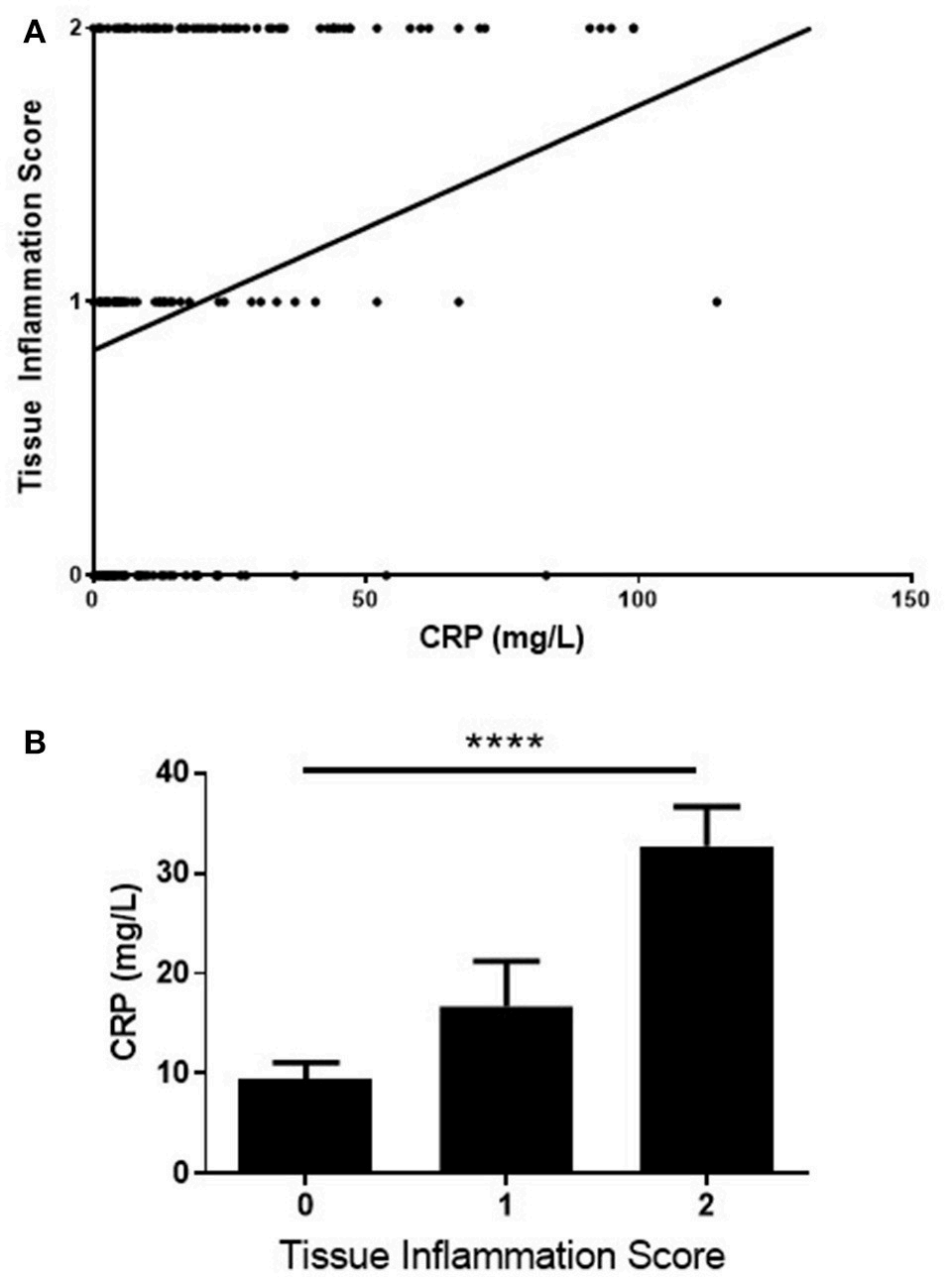
Correlation of CRP with tissue inflammation scores **(A)** correlation of CRP levels with tissue inflammation score on H+E staining. *n* = 197, rho = 0.43, Cl 0.30–0.54, ^****^p < 0.0001. **(B)** comparison of the CRP levels across the 3-point tissue inflammation score using Kruskaii-Wallis test, *p* = 0.0002.

**Figure 3:**
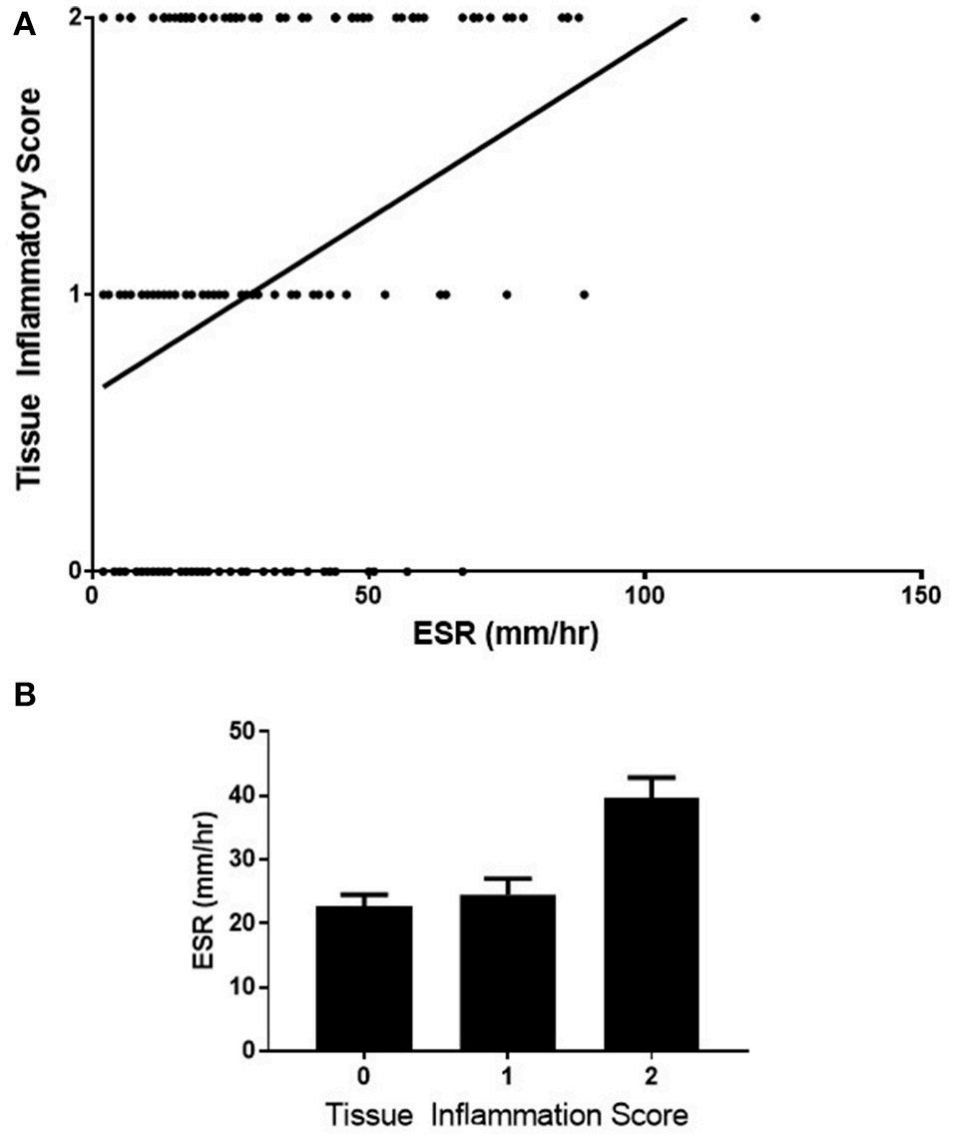
Correlation of ESR with tissue inflammation scores **(A)** correlation of ESR with tissue inflammation score on H+E staining. *n* = 188, rho = 0.29, Cl 0.15–0.42 *p* < 0.0001. **(B)** comparison of the ESR across the 3-point tissue inflammation score using Kruskaii-Wallis test, *p* = 0.0001.

A statistically significant positive correlation was observed between CRP and the level of inflammation in the biopsy retrieved (*n* = 197, rho = 0.43, CI 0.30–0.54, *p* < 0.0001), Figure 2A. To underline the robustness of these findings, this data was also examined using Kruskall-Wallis, for differences in CRP levels across each of the three tissue inflammatory scores (Figure 2B, *p* < 0.0001). A positive correlation was also observed between ESR and the level of inflammation in the biopsy retrieved, Figure 3A (*n* = 188, rho = 0.29, CI 0.15–0.42 *p* < 0.0001), and differences in ESR across the three Tissue Inflammation Scores were demonstrated using Kruskall-Wallis, Figure 3B, (*p* < 0.0001).

**Figure 4:**
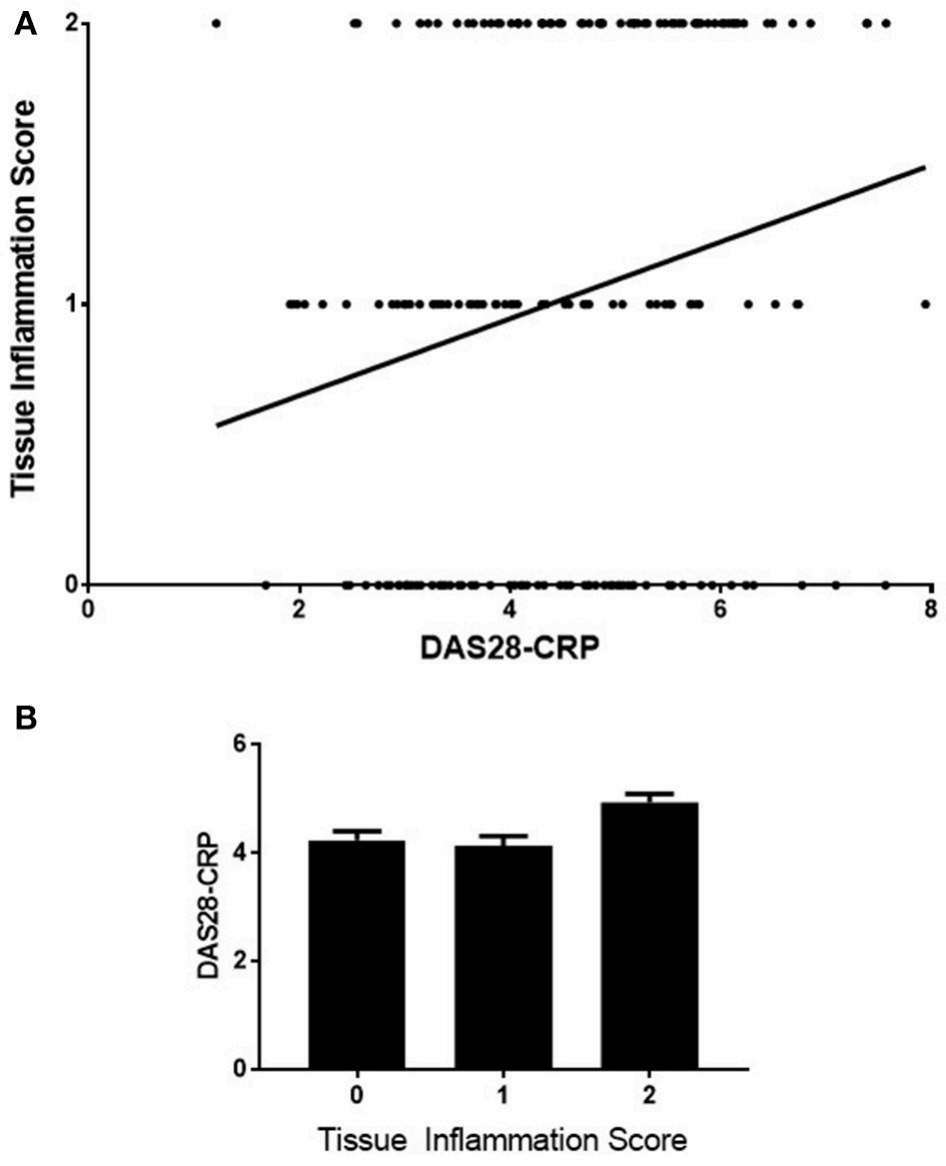
Correlation of DAS28-CRP with tissue inflammation scores **(A)** correlation of DAS28-CRP with tissue inflammation score on H+E staining (*n* = 189, rho = 0.23, Cl 0.09–0.37, *p* = 0.0011). **(B)** comparison of the DAS28-CRP levels across the 3-point tissue inflammation score using One-Way ANOVA test, *p* = 0.0009.

A statistically significant but weak positive correlation was observed between the DAS28-CRP and synovial inflammation (*n* = 189, rho = 0.23, CI 0.09–0.37, *p* = 0.0011), Figure 4A. Again, differences in DAS28-CRP across the three Tissue Inflammation Scores were observed using One Way ANOVA, Figure 4B (*p* = 0.0009).

#### Normal CRP and DAS28-CRP remission

**Figure 5:**
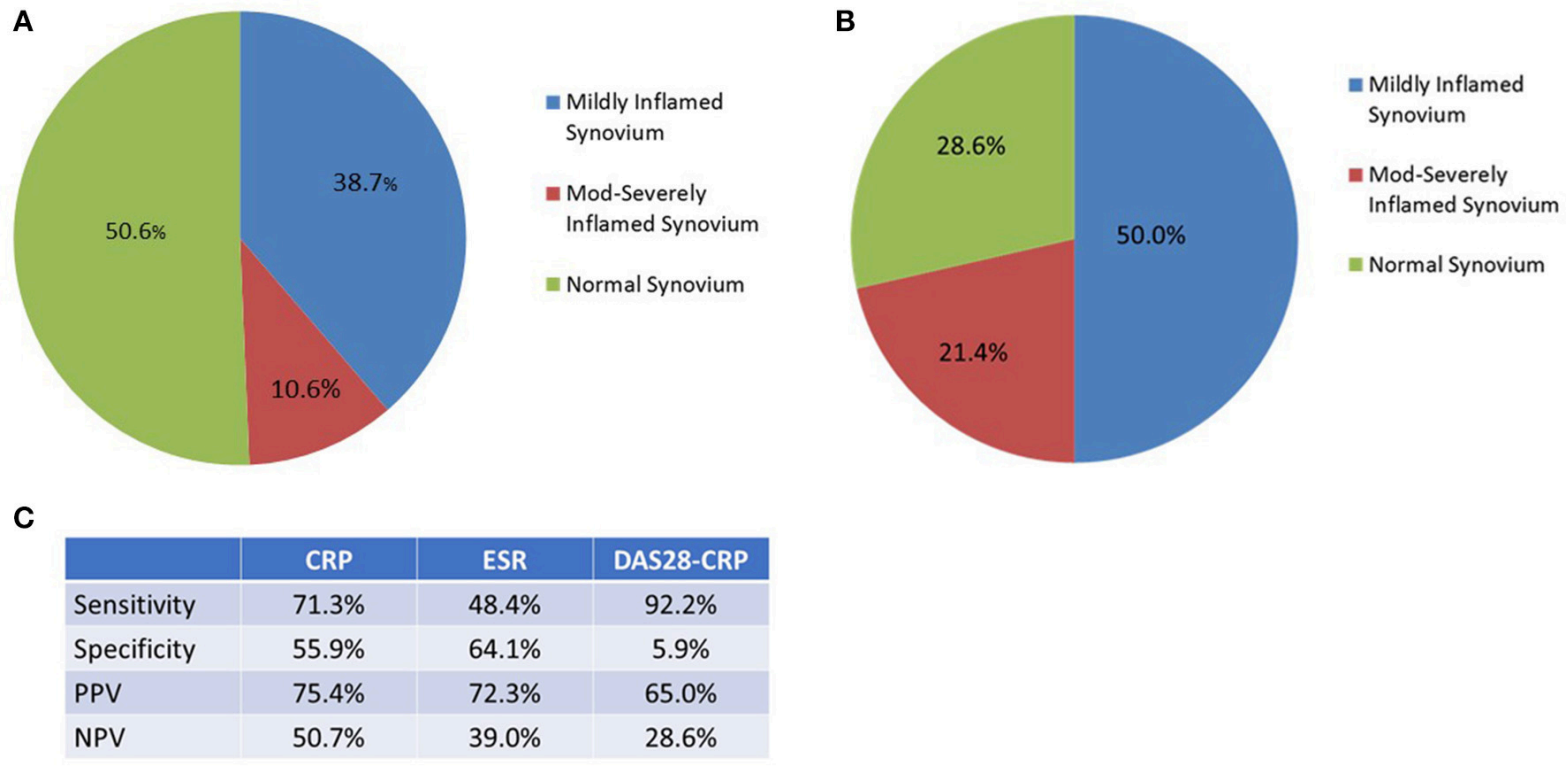
**(A)** In those with a normal CRP (<5 mg/L) at time of biopsy (*n* = 75), 49.4% had histological evidence of inflammation in the synovial tissue retrieved. **(B)** In those with a DAS28-CRP (< 2.6) at time of biopsy (*n* = 14), 71.4% had histological evidence of inflammation in the synovial tissue retrieved. **(C)** A comparison of the performance of CRP, ESR, and DAS28-CRP in identifying histological inflammation.

75 patients had a normal CRP (< 5 mg/L) on the morning of arthroscopy guided synovial biopsy. Of these, there was histological evidence of inflammation in 49.4%, Figure 5A. 14 patients were in DAS28-CRP remission (< 2.6) at time of biopsy, and again, 71.4% of their biopsies had evidence of inflammation, Figure 5B. The DAS28-CRP had the best performance in sensitivity to detect histological inflammation, but performed poorly in specificity. A comparison in performance of the variables studied in the detection of histological synovial inflammation is in Figure 5C.

#### Relationship of CRP to tissue immune markers

**Figure 6:**
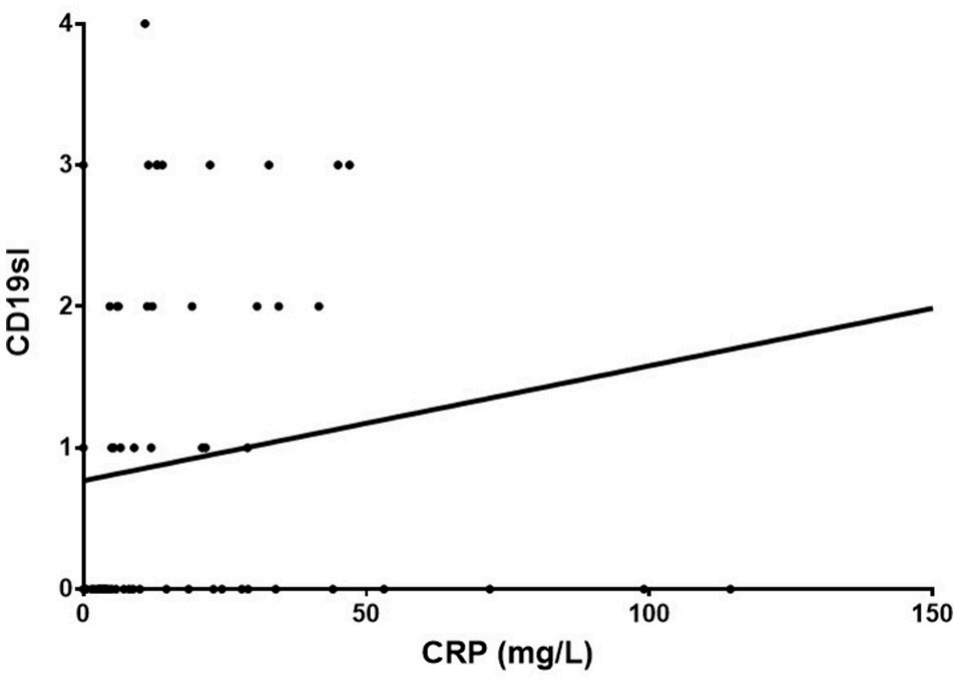
Correlation between CRP and CD19+ cellular infiltrate in the synovial sublining (*n* = 70, rho = 0.32, Cl 0.08–0.52, *p* = 0.0068).

A statistically significant correlation was observed between CRP and CD19+ cellular infiltrate in the synovial sublining (*n* = 70, rho = 0.32, CI 0.08–0.52, *p* = 0.0068), Figure 6. There was also a weak positive correlation at borderline significance between CRP and CD68+ (*n* = 115, rho = 0.18, CI −0.01 to 0.36, *p* = 0.055) and CD8+ (*n* = 76, rho = 0.22, CI −0.01 to 0.43, *p* = 0.055) cellular infiltrates, data not shown. No correlation was identified for CRP with CD3+, CD4+, or FVIII staining. Apart from a more replete representation of CD8 cells in the sublining of the synovium of those with abnormal CRP's (*n* = 76, 25 with normal CRP, 51 with abnormal CRP, *p* = 0.01015, data not shown), no differences across all other immune markers were noted.

## Discussion

By studying the target tissue of RA, in a large cohort of patients reflective of RA in the clinics, we have demonstrated that there is a clear positive correlation between CRP, ESR and DAS28-CRP with histological inflammatory changes in synovial biopsies. However, in relation to both CRP, and the DAS28-CRP, there is a lack of sensitivity in detecting inflammation in large minority of patients.

The features of the synovium where inflammation was present in those with both normal CRP's and abnormal CRP's were comparable, each comprising a mixture of both innate and adaptive immune cells. Therefore, despite arthralgia, this finding suggests an autoimmune etiology in both groups, and not inflammatory changes related to osteoarthritis. Furthermore, the severity of inflammation observed was not insignificant, with 10 and 20% in the normal CRP and DAS28-CRP remission categories having moderate to severe levels of inflammation respectively.

It is plausible that the patients falsely identified by CRP as being free of inflammation constitute a specific subset of RA, perhaps not identified by the IL6 mediated systemic inflammatory response. If so, it may be that alternative biomarkers of disease activity may be more appropriate. Not only will identifying such candidate biomarkers be a challenge, but identifying this cohort of RA patients in the first instance. Many alternative biomarkers of disease activity have been identified, including calprotectin, serum amyloid A, VEGF, VCAM-1 and many others. The difficulties with individual biomarkers has led to the development of a multi-biomarker disease activity (MBDA) algorithm, which uses 12 biomarkers to generate a score between 1 and 100 (21). Another possibility is that many RA phenotypes are not marked by the regular acute phase reactants because instead they have a greater contribution of other disease mechanisms, for example fibrosis (22). However, our study was directed toward the identification of inflammation, and it was in this context that CRP has failed to identify active disease in a cohort of RA patients. Further interrogation of the differing molecular disease networks within RA may assist in understanding why certain phenotypes are marked by elevations of a given marker during active disease, while others are not.

There are inherent problems with each of the currently available biomarkers in respect of disease activity. It is known, for example, that secretion of CRP is largely IL-6 and TNFα dependant, and novel therapies such as tocilizumab (23), tumor necrosis factor α inhibition (24) and indeed, tofacitinib, interrupt this pathway. This may lead to a decrease in CRP, which does not necessarily reflect a decrease in disease activity.

In contrast to the clinical research setting, how important recognizing a purist definition disease remission in the clinics is not clear. Remission should of course mean the absence of any disease activity at all, but definitions require both validity and feasibility, each of which may compromise the other. Boolean remission criteria are unpopular, but purposeful in the research setting. Agreement in definitions of remission did not include a temporal parameter, and it is now known that sustained remission in RA is uncommon(25). Arguably the most important criteria in the routine clinical setting is the DAS28-CRP, as it appraises a feasible number of joints, includes a widely available, relatively inexpensive, single biomarker, and takes account of the effect of disease on the patient based on their subjective report. For this reason, we selected the DAS28-CRP as the disease activity score used in this study. In addition, although inflammatory markers such as CRP exist on a spectrum, their interpretation in the clinical setting is often binary; normal/abnormal. Therefore, it is important to understand what the performance of markers are in this context.

This study has some limitations, and whether the findings should change clinical practice is debateable. It is not at all clear that treating “subclinical” synovitis is either feasible or desirable (26). Evidence from sonographic studies suggests that treating synovitis detected in this way does not change outcomes (27, 28), and the same may well be true in those with histological evidence of persistent inflammation. However, there is evidence that even in patients with sustained remission, progression of the disease in the form of the development of new erosions occurs in up to 33% of patients (29, 30). This data suggests either a lack of sensitivity of remission criteria in defining true remission, or an “uncoupling” of the inflammatory process itself from radiological evidence of persisting damage. Although the data we present suggests the former, at least for DAS28-CRP remission, it in no way precludes the latter from contributing to erosive disease also. Furthermore, all patients included in this study, including those with normal CRP and in remission by DAS28-CRP, were reporting arthralgia in the joint biopsied, and therefore these individuals may be more likely to have synovitis than others with the same CRP/DAS28-CRP.

There is reason to believe however, that the inflammatory changes seen in the synovium retrieved from the knee joints of the patients in this study, is representative of synovium from any other site. It has previously been shown that biopsies taken from an inflamed knee did not differ in mean cell numbers for all markers investigated when compared with contemporaneously retrieved small joint synovium (31). This data emphasizes the relevance of studying synovial tissue retrieved from knee joints in investigating RA. It also suggests that the observed inflammation in the tissue we have analyzed in our study, reflects a systemic inflammatory process (not exclusively localized to the knee joint). It is also notable that clinically uninvolved joints had similar histological abnormalities as those with overtly evident disease involvement (32, 33). Taken together, these studies support the hypothesis that what we have found in knee synovium, may be representative of a more systemic process involving any synovial joint.

## Conclusion

It is clear from this study, that while CRP has a moderately strong relationship with disease activity, there are significant pitfalls in the use of this biomarker in RA, and therefore a need interpret CRP results judiciously. Nevertheless, there clearly remains a role for this inexpensive and readily available biomarker in the evaluation of disease activity in the majority of those with RA. The results of this study underline the heterogeneity of RA. There is therefore an urgent need to develop better biomarkers, and to identify methodologies to determine at diagnosis, which patients will not be accurately appraised by CRP. The more holistic DAS28-CRP, suffers from similar pitfalls to CRP itself.

## Author contributions

CO, UF, and DV: Conception and design; CO, AN, FY, TM, and MB: Acquisition of data; CO, UF, and DV: Statistical analysis; CO, UF, and DV: Manuscript preparation.

### Conflict of interest statement

The authors declare that the research was conducted in the absence of any commercial or financial relationships that could be construed as a potential conflict of interest.
